# *Bifidobacterium bifidum* DS0908 and *Bifidobacterium longum* DS0950 Culture-Supernatants Ameliorate Obesity-Related Characteristics in Mice with High-Fat Diet-Induced Obesity

**DOI:** 10.4014/jmb.2210.10046

**Published:** 2022-11-22

**Authors:** M. Shamim Rahman, Youri Lee, Doo-Sang Park, Yong-Sik Kim

**Affiliations:** 1 Institute of Tissue Regeneration, College of Medicine, Soonchunhyang University, Cheonan 31151, Republic of Korea; 2 Department of Microbiology, College of Medicine, Soonchunhyang University, Cheonan 31151, Republic of Korea; 3 Biological Resource Center, Korea Research Institute of Bioscience and Biotechnology, Jeongeup 56212, Republic of Korea

**Keywords:** Anti-obesity, probiotics, postbiotics, thermogenesis, PKA/p38 MAPK

## Abstract

Probiotic supplements have promising therapeutic effects on chronic diseases. In this study, we demonstrated the anti-obesity effects of two potential probiotics, *Bifidobacterium bifidum* DS0908 (DS0908) and *Bifidobacterium longum* DS0950 (DS0950). Treatment with DS0908 and DS0950 postbiotics significantly induced the expression of the brown adipocyte-specific markers UCP1, PPARγ, PGC1α, PRDM16 and beige adipocyte-specific markers CD137, FGF21, P2RX5, and COX2 in C3H10T1/2 mesenchymal stem cells (MSCs). In mice with high-fat diet (HFD)-induced obesity, both potential probiotics and postbiotics noticeably reduced body weight and epididymal fat accumulation without affecting food intake. DS0908 and DS0950 also improved insulin sensitivity and glucose use in mice with HFD-induced obesity. In addition, DS0908 and DS0950 improved the plasma lipid profile, proved by reduced triglyceride, low-density lipoprotein, and cholesterol levels. Furthermore, DS0908 and DS0950 improved mitochondrial respiratory function, confirmed by the high expression of oxidative phosphorylation proteins, during thermogenesis induction in the visceral and epididymal fat in mice with HFD-induced obesity. Notably, the physiological and metabolic changes were more significant after treatment with potential probiotic culture-supernatants than those with the bacterial pellet. Finally, gene knockdown and co-treatment with inhibitor-mediated mechanistic analyses showed that both DS0908 and DS0950 exerted anti-obesity-related effects via the PKA/p38 MAPK signaling activation in C3H10T1/2 MSCs. Our observations suggest that DS0908 and DS0950 could potentially alleviate obesity as dietary supplements.

## Introduction

Probiotics are a group of beneficial gut microorganisms that reportedly alleviate several chronic diseases, such as obesity, cancer, cardiovascular disease, and type 2 diabetes [1, 2]. They are commonly found in infant gut microbiomes and improve an infant’s immunity [2]. They colonize the gastrointestinal tract and catabolize human milk oligosaccharides, a common source of prebiotics (*i.e.*, non-digestible food components that complement probiotic growth) for their growth [3]. They produce several postbiotics (*i.e.*, short-, medium- and long-chain fatty acids) and secondary metabolites [4]. Probiotics reportedly interact directly with human intestinal epithelial cells, enhancing anti-inflammatory responses [4, 5]. Furthermore, postbiotics (*e.g.*, short-chain fatty acids) reportedly improve energy metabolism and adiposity [6-8]. One of the striking features of postbiotics is the ability to penetrate the blood-brain barrier and regulate sustainable energy homeostasis by improving insulin or leptin receptor signaling, achieved via G-coupled protein receptor (GPR) signaling modulation [9, 10]. Recent studies show that sustainable energy metabolism improvement is an attractive therapeutic approach for obesity and co-morbidities [11-14].

Obesity is characterized by the imbalance of energy preservation and expenditure in metabolic systems, mainly those of the body fat cells; white, brown and the newly discovered beige (or brite) adipocytes. The relatively lipid-laden white adipocytes contain fewer mitochondria and contribute to obesity by hypertrophy, lipid content increase, or hyperplasia. Adipokines secreted by white adipocytes cause normal metabolic dysfunction and several metabolic diseases [15-17]. In contrast, brown adipocytes ameliorate obesity by inducing energy expenditure. They activate mitochondrial oxidative phosphorylation and uncoupling protein 1 (UCP1), a mitochondrial inner membrane protein [16]. Brown adipocytes exhibit multi-locular lipid droplets potentially generated during lipolysis in the mitochondrial fat β-oxidation process, as reported in multiple studies [18, 19]. Also, the newly discovered beige adipocytes share features similar to the brown adipocytes commonly found in white-fat deposits. Beige adipocytes originate both from white adipocytes (undergoing a process called browning) and adipogenic progenitors [18-22]. Therefore, discovery of potential therapeutic candidates to induce brown or beige adipocyte activity has emerged as a popular obesity treatment option.

Several signaling (*e.g.*, AMPK, PKA, p38 MAPK, and CREB) and regulatory (*e.g.*, PPARγ, PRDM16, PGC1α, and UCP1) proteins are involved in the induction of white adipocyte transdifferentiation into brown or beige adipocytes [8, 19, 21, 22]. During thermogenic induction, phosphorylation of signaling proteins activates the underlying transcription factor cascade. Peroxisome proliferator-activated receptor gamma (PPARγ), also known as the master adipogenesis regulator, exhibits browning regulatory effects in white adipocytes [20, 23]. PR-domain containing protein 16 (PRDM16) reportedly improves brown and beige adipocyte activities [24-26]. PPARγ-coactivator-1α (PGC1α) regulates mitochondrial biogenesis and energy homeostasis by affecting the electron transport chain (ETC), thereby enhancing mitochondrial oxidative phosphorylation (OXPHOS) [27, 28]. The free fatty acids generated from lipid β-oxidation are converted to oxidized electrons, which are carried through the ETC. It forms a complex with UCP1 to produce cellular ATP, utilized during energy scarcity [29].

Recent studies have shown that probiotics exhibit significant potential to mitigate obesity. Certain strains of a common probiotic species, *Bifidobacterium*, are widely used as dietary supplements and functional foods, and reportedly improve metabolic function and protect against dysmetabolic diseases. For example, *Bifidobacterium infantis*, one of the most abundant gut microbes in breastfeeding infants, displays the ability to improve infant immunity, including the induction of protective antibody production, and directly affects adipocyte physiology [12, 30, 31]. A lower count of *Bifidobacterium* species in the gut microbiome increases the risk of childhood obesity [12]. *B. infantis* catabolizes human milk oligosaccharides, improving UCP1 and leading to abundant multi-locular lipid droplet levels in adipocytes [12]. We have previously demonstrated that *B. infantis* reduces lipid accumulation and induces browning in white adipocytes [7, 8]. In this study, we describe how *B. bifidum* DS0908 and *B. longum* DS0950 can induce thermogenesis under both in vitro and in vivo conditions, and may have significant therapeutic potential against obesity.

## Materials and Methods

### Bacteria Isolation and Identification

Two *Bifidobacterium* strains, *B. bifidum* DS0908 and *B. longum* DS0950, were isolated from 1 g of human fecal sample diluted in 10 ml of sterilized 0.85% PBS solution. Serially diluted fecal supernatants were spread on De Man, Rogosa and Sharpe (MRS) agar medium (Becton, Dickinson and Company, USA) and incubated in an anaerobic chamber (Coy Laboratory Products, USA) filled with 5% H_2_, 10% CO_2_ and 85% N_2_ at 37°C. After 48 h, a single ivory colony was obtained and identified by 16S rRNA sequencing. For 16S rRNA sequencing, the bacterial genomic DNA was extracted as described previously [32]. The 16S rRNA gene sequence was amplified using the universal primers 27F (5'-AGAGTTTGATCMTGGCTCAG-3') and 1492R (5'-TACGGYTACCTTGTTACGACTT-3'). To ascertain the phylogenetic position of the isolates, we compared the 16S rRNA gene sequences with sequences obtained from the EzBioCloud (https://www.ezbiocloud.net/) and GenBank/EMBL/DDBJ (http://www.ncbi.nlm.nih.gov/blast) databases, respectively. The bacterial strains were deposited in the Korean Collection for Type Cultures (KCTC 13935BP for *B. bifidum* DS0908 and KCTC 13936BP for *B. longum* DS0950).

### Probiotic Culture-Supernatant Preparation

Bacterial cells were cultured on MRS agar at 37°C in an anaerobic chamber. After 48 h, a single colony was picked, seeded onto 30 ml of fresh MRS medium and grown for 36 h at 37°C under anaerobic conditions. The liquid medium was purged with ultra-pure nitrogen gas for 15 min before autoclaving to achieve anaerobic conditions. The residual oxygen was removed by keeping the medium air-permeable in an anaerobic chamber. The oxygen concentration was controlled to 0 ppm when measured with a CAM-12 anaerobic monitor (Coy Laboratory Products). Culture supernatants were collected after centrifugation of the microbial culture (CFU; 5 × 10^9^ cells/ml) at 11,000 ×*g* for 20 min, then freeze-dried and dissolved in 1/10^th^ volume of distilled water (v/v). The culture supernatants were stored at −20°C until further use. *B. bifidum* DS0908 and *B. longum* DS0950 culture supernatants (postbiotics and secondary metabolites) were selected using qRT-PCR based *Ucp1*-screening assay as described previously [8]. According to J. Prasad *et al*., our studied bacterial strains adhere to the requirements of potential probiotics [33]. The probiotic bacterial strains used in the screening assay are listed in Table S1.

### Reagents and Antibodies

Insulin, dexamethasone, 3-isobutyl-1-methylxanthine (IBMX), rosiglitazone (Rosi), H89, SB 203580, 8-br-cAMP, 4% formaldehyde, and dimethyl sulfoxide were purchased from Sigma-Aldrich (USA). Fetal bovine serum (FBS) and high-glucose Dulbecco’s modified Eagle medium (DMEM) were purchased from Atlas Biologicals (USA). Penicillin-streptomycin solution was acquired from Hyclone Laboratories, Inc. (USA). Antibodies against UCP1, PGC1α, PRDM16 and OXPHOS proteins were purchased from Abcam (USA). Antibodies against *aP2*, AMPK, p-AMPK (Thr172), p38 MAPK, p-p38 MAPK (Thr180/Tyr182), p-CREB (Ser133), p-PKA substrate and anti-rabbit IgG anti-mouse IgG antibodies conjugated with horseradish peroxidase were acquired from Cell Signaling Technology (USA). Antibodies against PPARγ and β-actin were purchased from Santa Cruz Biotechnology (USA). The BCA Protein Assay Kit was acquired from Thermo Fisher Scientific (USA) and the protein loading buffer from Bio-Rad Laboratories, Inc. (USA).

### Cell Culture

C3H10T1/2 MSCs (KCLB-10226; passage number ≥ 10) were cultured in DMEM GlutaMax supplemented with 10% FBS and 1% penicillin-streptomycin solution in a humidified 5% CO_2_ incubator at 37°C. For adipogenic differentiation, sufficiently confluent C3H10T1/2 MSCs (2 days post confluence, designated as day 0) were incubated with an adipogenic differentiation cocktail MDI (0.5 mM IBMX, 1 μM dexamethasone and 10 μg/ml insulin), with or without DS0908 and DS0950 in DMEM supplemented with 10% FBS. Treatments exceeding two days were continued until day 4, and DS0908 and DS0950 were added to the maturation medium containing DMEM, insulin and 10% FBS for cell culture on days 3 and 4. After day 4, only the maturation medium was used until harvest. Fully differentiated C3H10T1/2 MSCs were harvested on day 6 and used for the study. Incubation in MDI served as a negative control, and incubation with Rosi (1 μM) in DMEM was a positive control.

### Animal Studies

Specific-pathogen-free (SPF) male C57BL/6 mice (6 weeks old) were purchased from Koatech (Korea) and maintained at 22°C ± 2°C under 40–60% relative humidity and a 12:12 h, light-dark cycle for one week to stabilize their metabolism. The mice were fed a normal-fat diet (NFD, D12450B, Research Diets Inc., USA) or a high-fat diet (HFD) (D12492, Research Diets Inc.) for four weeks to generate mice with HFD-induced obesity before DS0908 or DS0950 administration. Then, the mice were separated into seven groups of 8 individuals (n = 8) as follows: G1 (NFD), G2 (HFD), G3 (HFD + BS [culture supernatants of DS0908]), G4 (HFD + BS [culture supernatants of DS0950]), G5 (HFD + bacterial pellet (BP) of DS0908]), G6 (HFD + BP [bacterial pellet of DS0950]) and G7 (HFD + Rosi). The mice were administered DS0908 or DS0950 pellets (1 × 10^9^ cells/kg) or culture supernatants (150 ml/mouse) by oral gavage for five days a week for eight weeks. Body weight and food intake were recorded once and three times a week. After six weeks of administration, we randomly selected 4 mice from each group and mice were fasted for 16 h and 5 h for oral glucose tolerance test (OGTT) and insulin tolerance test (ITT), respectively. Before injection, we collected blood as a baseline control (0 min). After glucose (OGTT) or insulin (ITT) injection, we measured blood glucose levels at 15, 30, 60, 90, and 120 min. The liver, as well as subcutaneous, epididymal, and mesenteric fat were then surgically removed, weighed, immediately frozen in liquid nitrogen, and immersed in RNAlater (Thermo Fisher Scientific), or fixed in 10% neutral formalin. The animal use and care protocol for this experiment was approved by the Institutional Animal Care and Use Committee at Korea Research Institute of Bioscience and Biotechnology (KRIBB) (approval no. KRIBB-AEC-20093).

### Haematoxylin and Eosin (H&E) Staining

The tissues (epididymal fat and liver) were fixed in 4% paraformaldehyde, embedded in paraffin, and cut into 10-μm sections. Standard H&E staining was performed using standard protocols. Five random fields of each section were evaluated, and the average adipocyte diameter was measured using the ImageJ software (NIH, USA).

### Quantitative RT-PCR Analysis

We harvested C3H10T1/2 MSCs and extracted total RNA using an RNA Extraction Kit (Qiagen, USA) following the manufacturer’s guidelines. We used 1 μg RNA to synthesize cDNA using the Maxime RT PreMix Kit (Intron Biotechnology, Korea) on a Veriti 96-Well Thermal Cycler (Applied Biosystems, Singapore). qRT-PCR was performed using an iQ SYBR Green Supermix Kit (Bio-Rad, Singapore) on a CFX96 Real-Time PCR Detection System (Bio-Rad). *Tbp* was an internal control. Table 1 contains the sequence information of the primers.

### Western Blot Analysis

Differentiated and treated C3H10T1/2 MSCs were prepared in RIPA lysis buffer supplemented with protease inhibitors. The total protein concentration was measured using the BCA Protein Assay Kit (Thermo Fisher Scientific). Equal amounts of protein samples were separated on a 4–20% sodium dodecyl sulfate-polyacrylamide gradient gel (Mini-PROTEAN Precast Gel, Bio-Rad) and transferred onto polyvinylidene difluoride (PVDF) membranes. The PVDF membranes were then blocked and incubated with specific antibodies, as indicated in the figures. Immunoreactive protein bands were captured using the chemiluminescent ECL (Advansta Inc., USA) assay on ChemiDoc XRS^+^ with ImageLab (Bio-Rad). Anti-β-actin antibody was used as a loading control for each protein expression. Protein band intensities were quantified using the ImageJ software.

### PKA and p38 MAPK Knockdown Studies

C3H10T1/2 MSCs were seeded on 6-well plates and grown to 80–90% confluence. The cells were then transfected with control siRNA (siCont, 50 nM), PKAα siRNA (siPKA, 50 nM) or p38 MAPKα siRNA (sip38, 50 nM) oligonucleotide duplexes (Santa Cruz Biotechnology, Inc.) using Lipofectamine 2000 (Invitrogen, USA) according to the manufacturer’s instructions. Transfection efficiency was determined using qRT-PCR and western blotting.

### Statistical Analysis

All values are expressed as the average ± standard error mean (SEM). The experimental determinants were confirmed by using at least triplicate biological samples. Student’s *t*-test or two-way ANOVA (OGTT, ITT, body weight gain, and food intake) were used to distinguish the statistical significance levels, where *, **, ***, and ns indicates *p* < 0.05, < 0.01 and < 0.001, and no significance, respectively.

## Results

### Treatment with DS0908 and DS0950 Induces Thermogenesis in C3H10T1/2 MSCs and in Mice with HFD-Induced Obesity

**Fig. 1:**
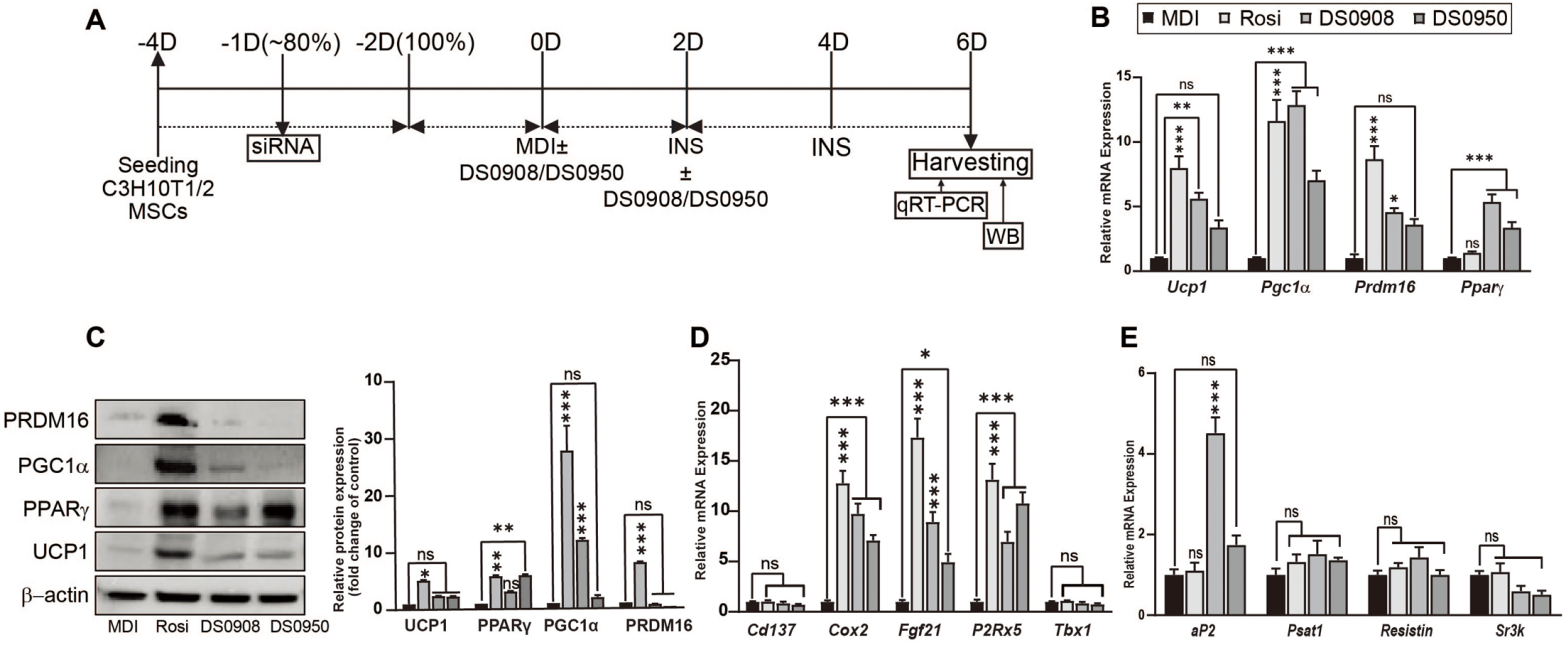
Effect of *B. bifidum* DS0908 (DS0908) and *B. longum* DS0950 (DS0950) treatment on thermogenesis in C3H10T1/2 MSCs. (**A**) Schematic representation of C3H10T1/2 MSCs differentiation timeline. (**B**) mRNA expression levels of the major thermogenic markers *Ucp1*, *Pgc1α*, *Prdm16* and *Pparγ* after DS0908 and DS0950 treatment in differentiated C3H10T1/2 mesenchymal stem cells (MSCs). (**C**) Thermogenic marker UCP1, PPARγ, PGC1α and PRDM16 protein expression levels after DS0908 and DS0950 treatments in differentiated C3H10T1/2 MSCs. (**D** and **E**) mRNA expression levels of beige (*Cd137*, *Fgf21*, *P2rx5* and *Tbx1*), brown (*Cox2*) and white (*aP2*, *Psat1*, *Resistin* and *Serpina3k*) adipocyte-specific markers after DS0908 and DS0950 treatments in differentiated C3H10T1/2 MSCs. *Tbp* was used as an internal control gene and β‐actin as a protein loading control. The data from three individual experiments are expressed as the average ± standard error mean (SEM). *, **, *** and ns indicate *p* < 0.05, < 0.01, < 0.001 and non-significant, respectively, to express the statistically significant differences between the control (MDI) and the treatment groups in the figures. The protein band intensities were measured using ImageJ. Adipogenic differentiation medium, MDI: 0.5mM IBMX, 1 μM dexamethasone and 10 μg/ml insulin; 1 μM Rosiglitazone (Rosi); DS0908 = *B. bifidum* DS0908; DS0950 = *B. longum* DS0950.

Our previous study showed that *B. bifidum* DS0908 (DS0908) and *B. longum* DS0950 (DS0950) promote brown adipocyte-, beige adipocyte-, and lipolysis-related marker expressions in 3T3-L1 pre-adipocytes. Treatment with culture supernatants of DS0908 and DS0950 reduced intracellular triglyceride (TG) accumulation during thermogenesis induction, confirmed by ORO staining and TEM analysis [8]. These findings prompted us to evaluate the thermogenic effect of DS0908 and DS0950 in vitro (Fig. 1A). To investigate the thermogenic effect in DS0908- and DS0950-treated C3H10T1/2 MSCs, we examined common thermogenic marker expressions in 5 μl/ml DS0908- and DS0950-treated C3H10T1/2 MSCs (Fig. 1B). Both the DS0908 and DS0950 treatments increased *Ucp1* (5.60- and 3.38-fold), *Pgc1α* (12.85- and 7.02-fold) and *Prdm16* (4.56- and 3.59-fold) mRNA expressions and UCP1 (2.41- and 2.32-fold), PPARγ (3.12- and 5.93-fold), PGC1α (12.06- and 1.98-fold) and PRDM16 (0.70- and 0.31-fold) protein expressions in C3H10T1/2 MSCs (Figs. 1B and 1C). The PRDM16 protein expression levels were lower in the DS0908 and DS0950 treatment groups than the control. The DS0908 and DS0950 treatments also significantly increased the mRNA expression of beige adipocyte-specific markers *Fgf21* (8.94- and 4.94-fold) and *P2rx5* (6.96- and 10.80-fold), as well as the brown adipocyte-specific marker *Cox2* (9.76- and 7.10-fold) in C3H10T1/2 MSCs. However, we observed no significant change in the *Cd137* and *Tbx1* mRNA expression levels (Fig. 1D). Next, we evaluated the mRNA expression of the common white adipocyte markers *Serpina3k*, *Psat1* and *Resistin*, as well as adipogenesis-related markers *aP2* (4.51- and 1.74-fold), *Psat1* (1.51- and 1.36-fold), *Resistin* (1.43- and 1.00-fold) and *Serpina3k* (0.59- and 0.51-fold) in DS0908 and DS0950-treated C3H10T1/2 MSCs (Fig. 1E). We observed no distinguishable change in the expression of white adipocyte markers, where adipogenic differentiation markers were significantly induced upon DS0908 and DS0950 treatment. To reaffirm these findings in vivo, we examined common thermogenic markers in the visceral and epididymal fat in the DS0908 and DS0950 treatment groups (both BS and BP). We observed that DS0908 and DS0950 culture supernatants increased *Ucp1* (137.37- and 124.96-fold), *Prdm16* (25.60- and 267.22-fold) and *Ppar*γ (0.07- and 16.08-fold), as well as the beige adipocyte-specific marker *Cd137* (0.85- and 5.90-fold) mRNA expressions (Fig. S1A). In addition, the *aP2* expression remained unchanged in both treatment groups. As with gene expression, distinguishable changes also appeared in the UCP1, AP2, PGC1α, PPARγ and PRDM16 protein expressions upon treatment with DS0908 and DS0950 culture supernatants, both in the visceral and epididymal fat tissues (Figs. S1B and 1C). DS0908 and DS0950 supplementation (both BS and BP) in mice with HFD-induced obesity improved mitochondrial oxidative capacity, supported by the OXPHOS protein expressions. Along with our previous findings in 3T3-L1 preadipocytes [8], these results suggest that the DS0908 and DS0950 treatment can induce thermogenesis in C3H10T1/2, 3T3-L1 preadipocytes, and in mice with HFD-induced obesity.

### Treatment with DS0908 and DS0950 Reduces Weight Gain and Fat Accumulation without Altering Food Intake in Mice with HFD-Induced Obesity

**Fig. 2:**
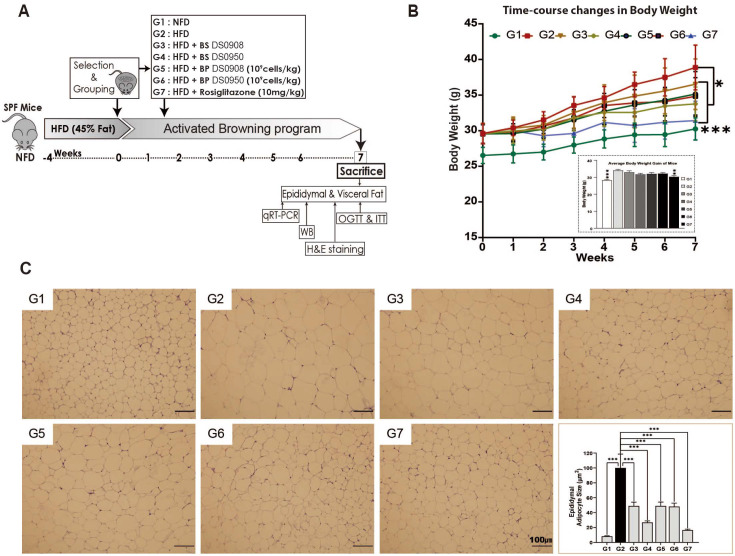
DS0908 and DS0950 supplementation reduce bodyweight and fat accumulation without altering food intake in mice with HFD-induced obesity. (**A**) Illustration of the dietary intervention timeline of mice with high-fat diet (HFD)-induced obesity and treatment group designs (*n* = 8 mice/per group). (**B**) Change in body weight growth curve with the average of whole-body weight gain after seven weeks in DS0908- and DS0950-administered mice with HFD-induced obesity compared to control mice group (NFD). (**C**) Haematoxylin and eosin (H&E) staining images of epididymal adipose tissue and lipid droplet area quantification among tissues from different mouse groups. The data are expressed as the average ± standard error mean (SEM). *, **, *** and ns indicate *p* < 0.05, < 0.01, < 0.001 and non-significant, respectively, to express the statistically significant differences between the control (G2: HFD) and treatment groups. G1: Normal-fat diet (NFD); G2: High-fat diet (HFD); G3: BS DS0908 (DS0908); G4: BS DS0950 (DS0950); G5: BP DS0908 (DS0908; 10^9^ cells/kg); G6: BP DS0950 (DS0950; 10^9^ cells/kg); G7: Rosiglitazone (Rosi; 10 mg/kg); BS = bacterial supernatant; BP = bacterial pellets.

The thermogenic and lipid-lowering potential of DS0908 and DS0950 culture supernatants prompted us to examine how they could potentially affect HFD-induced obesity in mice. To evaluate the DS0908 and DS0950 administration effect on mice with HFD-induced C57BL/6 obesity, we randomly assigned eight C57BL/6 mice (*n* = 8) into seven groups as follows: NFD; (G1), HFD; (G2), HFD + BS (cell-free supernatant) of DS0908 (G3), HFD + BS (cell-free supernatant) of DS0950 (G4), HFD + BP (bacterial pellets) of DS0908 (G5), HFD + BP (bacterial pellets) of DS0950 (G6), and HFD + Rosiglitazone (G7). The HFD groups were fed a 45% HFD for four weeks to induce obesity in the animals (Fig. 2A). The mice with HFD-induced obesity were then administered DS0908 or DS0950 culture supernatants or pellets. In DS0908- and DS0950-administered obese mice, food intake and body weight were monitored for up to seven weeks. We observed no significant change in food intake in DS0908- and DS0950-administered (both cell-free probiotic supernatants and pellets) obese mice compared to the control HFD group (Fig. S2A). However, the overall weight gain in DS0908- and DS0950-supplemented obese mice was not distinguishable from that in the HFD-fed group after seven weeks. The HFD group gained significant body weight compared to the NFD group throughout the seven weeks (Fig. 2B). In contrast, DS0908 and DS0950 supplementation (with both BP and BS) in C57BL/6 obese mice resulted in a significant body weight gain reduction throughout the seven weeks (Fig. 2B). In addition, we evaluated the effect of DS0908 and DS0950 culture supernatants and bacterial pellets of epididymal adipocyte size after H&E staining. We observed that the HFD group showed a larger adipocyte size than the NFD and Rosi (positive control) groups (Fig. 2C). Interestingly, adipocyte size in the case of the known thermogenesis-inducer rosiglitazone was similar to that in the NFD group. However, DS0908 and DS0950 supplementation (both with BP and BS) in HFD-induced C57BL/6 obese mice markedly reduced adipocyte size compared to that in the HFD-fed group. Adipocyte size in the DS0950 culture supernatant-supplemented group was smaller than that in the HFD group and similar to that in the Rosi-administered group (Fig. 2C). Next, we measured the common lipid-accumulated fat depot weights. DS0908 and DS0950 supplementation (both with BP and BS) slightly reduced epididymal, visceral, and subcutaneous fat, as well as liver weight compared to the HFD group (Fig. S2B). Interestingly, these fat depot weights were significantly reduced in the Rosi-administered group compared to those in the HFD-fed group (Fig. S2B). These results indicated that the administration of both DS0908 and DS0950 effectively alleviated HFD-induced obesity by lowering body weight and adipocyte hypertrophy without affecting food intake in mice with HFD-induced obesity.

### Treatment with DS0908 and DS0950 Improves Insulin Sensitivity, Glucose Mmetabolism, and Lipid Profile in Mice with HFD-Induced Obesity

**Fig. 3:**
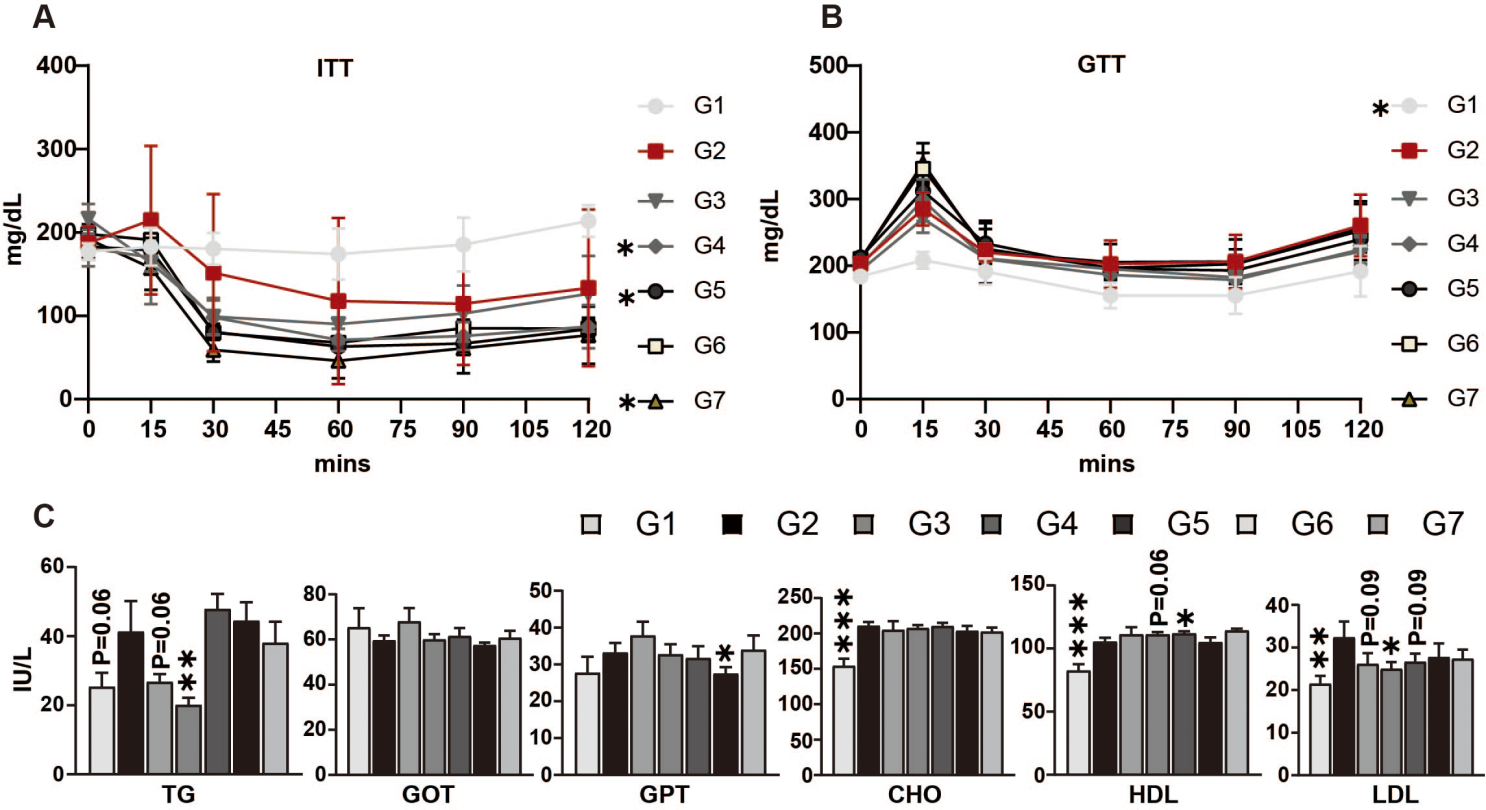
Effect of DS0908 and DS0950 supplementation on insulin tolerance, glucose utilization, and hormonal changes in mice with HFD-induced obesity. (**A** and **B**) Glucose and insulin uptake measurements in DS0908- and DS0950-administered mice with HFD-induced obesity. After six weeks, four mice from each group were selected and fasted for 16 h (OGTT) and 5 h (ITT), respectively. Before glucose (OGTT) or insulin (ITT) injection, blood was collected as a baseline control at 0 min, and then after glucose or insulin injection, blood glucose levels were measured at 15, 30, 60, 90, and 120 min. (**C**) Total triglyceride and cholesterol level (TG, HDL, and LDL) measurements and essential marker changes (GOT, GPT and CHO) in DS0908- and DS0950-administered mice with HFD-induced obesity. The data are expressed as the average ± standard error mean (SEM). *, **, ***and ns indicate *p* < 0.05, < 0.01, < 0.001 and non-significant, respectively, to express the statistically significant differences between the control (G2: HFD) and treatment groups. G1: Normal-fat diet (NFD); G2: High-fat diet (HFD); G3: BS DS0908 (DS0908); G4: BS DS0950 (DS0950); G5: BP DS0908 (DS0908; 10^9^ cells/kg); G6: BP DS0950 (DS0950; 10^9^ cells/kg); G7: Rosiglitazone (Rosi; 10 mg/kg); BS = bacterial supernatant; BP = bacterial pellets; TG = triglyceride; HDL = high-density lipoprotein; LDL = low-density lipoprotein; GOT = glutamic oxaloacetic transaminase; GPT = glutamic pyruvate transaminase; CHO = cholesterol.

To evaluate how DS0908 and DS0950 culture supernatants and bacterial pellets affect glucose metabolism and plasma lipid profiles, ITT and OGTT were performed after forty-four days of treatment. DS0908 and DS0950 supplementation (with BP and BS) noticeably reduced insulin levels, suggesting improved insulin sensitivity (Fig. 3A). Following the ITT, DS0908 and DS0950 supplementation (with BP and BS) markedly reduced glucose levels, implying that insulin uptake increased glucose use (Fig. 3B). Next, we determined how DS0908 and DS0950 supplementation (with BP and BS) impact the plasma lipid profile. We measured low-density lipoprotein (LDL), cholesterol (CHO), high-density lipoprotein (HDL), TG, aspartate aminotransferase/glutamic oxaloacetic transaminase (AST/GOT), and alanine aminotransferase/glutamic pyruvate transaminase (ALT/GPT) levels in the blood. We observed that DS0908 and DS0950 culture supernatant administration significantly reduced TG levels compared to those in the HFD and DS0908 and DS0950 pellet-treated groups (Fig. 3C). The AST/GOT expression remained unchanged between the HFD and DS0908- or DS0950-treated groups. In contrast, the ALT/GPT level was significantly lower in the DS0950 group (BP) (Fig. 3C). We also observed that DS0908 and DS0950 supplementation (both with BP and BS) lowered LDL and CHO levels while slightly increasing HDL levels in the blood. These findings suggest that DS0908 and DS0950 supplementation might ameliorate obesity by improving glucose metabolism and the plasma lipid profile in mice with HFD-induced obesity.

### Treatment with DS0908 and DS0950 Improves Thermogenesis via PKA/p38 MAPK Signaling in C3H10T1/2 MSCs

**Fig. 4:**
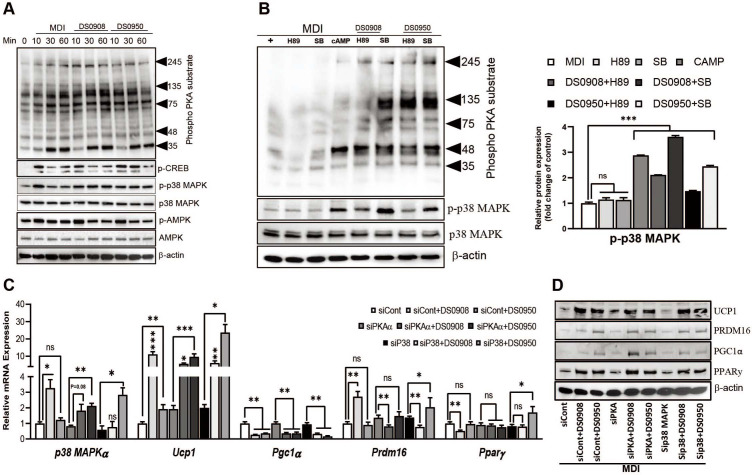
DS0908 and DS0950 culture supernatants activate thermogenesis via PKA-p38 MAPK signaling in C3H10T1/2 MSCs. (**A**) Phosphorylated protein expression levels of PKA substrates, p-p38 MAPK, p-CREB and p-AMPK after incubation with DS0908 and DS0950 for 10, 30, and 60 min. The confluent C3H10T1/2 MSCs were serum-depleted for 8 h, incubated with DS0908 and DS0950 for the indicated periods, then the phosphorylated proteins were detected. (**B**) Phosphorylation levels of PKA and p38 MAPK after treatment with 8-br-cAMP (PKA activator), H89 (pan-PKA inhibitor) and SB 203580 (p38 MAPK inhibitor) with or without DS0908 and DS0950. (**C**) mRNA expression levels of *p38 MAPKα* in *Pkaα* and *p38 MAPKα*-knockdown cells, and their downstream thermogenic genes *Ucp1*, *Pgc1α*, *Prdm16* and *Pparγ* after silencing of *Pkaα* and *p38 MAPKα* and treatment with DS0908 and DS0950 (siPkaα/sip38 MAPKα + DS0908 or DS0950 group) and control siRNA and treatment with DS0908 or DS0950 (siCont + DS0908 or DS0950 group). (**D**) Protein expression levels (UCP1, PGC1α, PRDM16 and PPARγ) were measured after silencing of *Pkaα* and *p38 MAPKα* and treatment with DS0908 or DS0950 (siPkaα/sip38 MAPKα + DS0908 or DS0950 group) and control siRNA and treatment with DS0950 or DS0908 (siCont + DS0908 or DS0950 group). The gene knockdown experiments were designed as siCont vs. siCont + DS0908 or DS0950, siPkaα vs. siPkaα + DS0908 or DS0950 and sip38 MAPKα vs. sip38 MAPKα + DS0908 or DS0950. Post silencing with the siRNA, C3H10T1/2 mesenchymal stem cells (MSCs) were differentiated as described in Methods. *Tbp* was used as an internal control gene and β‐actin as a protein loading control. The data from three individual experiments are expressed as the average ± standard error mean (SEM). *, **, *** and ns indicate *p* < 0.05, < 0.01, < 0.001 and non-significant, respectively, to express the statistically significant differences between the control (MDI) and the treatment groups in the figures. The protein band intensities were measured using ImageJ. Adipogenic differentiation medium, MDI: 0.5 mM IBMX, 1 μM dexamethasone and 10 μg/ml insulin; 1 μM Rosiglitazone (Rosi); DS0908 = *B. bifidum* DS0908; DS0950 = *B. longum* DS0950.

To understand the underlying mechanism of DS0908 and DS0950 culture supernatants related to thermogenesis, we examined the PKA, CREB, p38 MAPK and AMPK signaling pathways (Fig. S3A). First, we measured PKA and p38 MAPK phosphorylation after the DS0908 and DS0950 treatments. Both PKA and p38 MAPK phosphorylation markedly increased 60 min after the DS0908 and DS0950 treatments compared to MDI. However, the DS0908 and DS0950 treatments reduced AMPK and CREB^S133^ phosphorylation (Fig. 4A). These findings suggest that DS0908 and DS0950 culture supernatants might induce thermogenesis via PKA and p38 MAPK signaling in C3H10T1/2 MSCs. To confirm these findings, we performed a competitive chemical inhibition assay, co-treating DS0908 and DS0950 culture supernatants with H89 (a pan-PKA inhibitor) and SB 203580 (a p38 MAPK inhibitor). We observed that PKA and p38 MAPK phosphorylation decreased after the H89 and SB 203580 treatments, respectively. However, co-treatment with DS0908 and DS0950 culture supernatants recovered PKA and p38 MAPK phosphorylation. Notably, the p38 MAPK expression recovery was higher in DS0908 and DS0950 culture supernatant-related C3H10T1/2 MSCs (Fig. 4B). Under DS0908 and DS0950 culture supernatant co-treatment conditions, the H89 treatment reduced p38 MAPK expression. In contrast, PKA expression remained unaffected after the SB 203580 treatment, suggesting that PKA might be upstream of p38 MAPK. Furthermore, we performed PKAα or p38 MAPKα knockdown using small interfering RNA (siRNA)(Figs. 4C, 4D, and S3B). In PKAα-silenced cells, p38 MAPKα mRNA expression decreased by ~20%, whereas the DS0908 and DS0950 culture supernatant treatments markedly recovered the p38 MAPKα mRNA expression (1.82- and 2.11-fold). In contrast, the p38 MAPKα-silenced cells reduced the p38 MAPKα mRNA expression by ~50%, although the DS0908 and DS0950 culture supernatant treatments markedly increased the p38 MAPKα levels (0.77- and 2.83-fold, respectively) (Fig. 4C). These findings suggest that p38 MAPKα is downstream of the PKAα signaling. Next, we evaluated the downstream PKAα and p38 MAPKα thermogenic markers under siRNA knockout conditions. We observed that the *Ucp1*, *Pparγ*, and *Prdm16* mRNA expressions were markedly induced in siPKAα and sip38 MAPKα-knockdown cells following the DS0908 and DS0950 culture supernatant co-treatment (Fig. 4C). A similar expression pattern could also be observed at the thermogenic protein level (Figs. 4D and S3C). Overall, these data suggest that DS0908 and DS0950 promote thermogenesis via PKA/p38 MAPK signaling.

## Discussion

Obesity is associated with several chronic diseases, including cardiovascular disease, non-alcoholic fatty liver disease, diabetes, cancer, and severe COVID-19 [34, 35]. It begins with lipid content increase in white adipocytes, causing lipotoxicity and severe metabolic dysfunctions [16]. Therefore, the discovery of clinically significant anti-obesity therapeutics that reduce fat accumulation in white adipocytes has gained popularity over the past decades. Probiotics are dietary supplements used against several metabolic diseases [36]. In this study, we demonstrated the potential of two probiotic strains, *B. bifidum* DS0908 and *B. longum* DS0950, to reduce adiposity by inducing thermogenesis and inhibiting lipid accumulation both in vitro and in vivo.

At the onset of obesity, high lipid amounts accumulate in the white adipocytes related to fatty acid synthase, sterol regulatory binding protein 1c (SREBP1c), and acetyl-CoA carboxylase activity [8, 37, 38]. Upon lipid accumulation, white adipocytes expand both in size and number. Lipid-laden white adipocytes secrete several adipokines, such as adiponectin, resistin, and leptin. Overexpression of these adipokines reportedly causes insulin resistance and glucose metabolism disruption [39, 40]. High-fat content increases the TG, AST/GOT, ALT/GPT, CHO, and LDL levels, all of which are indicators of fatty liver disease [41]. Furthermore, an increased amount of mature white adipocytes increases body and organ weight and might affect food intake [8, 42, 43]. Multiple studies have reported that both culture supernatants and pellets of *B. infantis* reduce lipid accumulation in fat deposits by inhibiting adipo- and lipogenesis [7, 8, 11, 44]. The reduced fat accumulation level improves the plasma lipid profile by reducing the TG, AST/GOT, ALT/GPT, CHO and LDL levels and increasing those of beneficial HDL [7, 8, 11]. We also found that HFD feeding increased obesity-related marker expression in mice. However, *B. bifidum* DS0908 and *B. longum* DS0950 supplementation reduced epididymal adipocyte size and body and organ weights without altering food intake. It also improved the plasma lipid profile, insulin sensitivity, and glucose utilization.

Other approaches for ameliorating obesity include non-shivering thermogenesis and lipolysis activation in mature white adipocytes. Several studies demonstrated that *B. infantis* and its culture supernatants (also known as postbiotics encompassing short-, medium- and long-chain fatty acids) can induce thermogenesis by promoting white adipocyte browning to beige and by improving brown adipocyte activity [8, 9]. White-to-beige adipocyte conversion and adipogenic progenitor commitment to beige adipocytes are controlled by PPARγ, EBF2 and FGF21. *EBF2* and *FGF21* deletion blunt beige adipocyte formation, whereas their induction triggers thermogenesis in beige adipocytes [45, 46]. PPARγ plays a key role in adipogenesis, supporting white adipocyte maturation. However, treatment of white adipocytes with PPARγ agonists and adipogenic progenitors reportedly leads to beige and brown adipocyte formation [20, 21]. Both beige and brown adipocytes are characterized by high numbers of mitochondria and multi-locular lipid droplets. Mitochondria are considered the most important thermogenic process-related organelles. Mitochondria use electrons produced in free fatty acid lipid β-oxidation. While generating free fatty acids, lipolysis also produces small multi-locular lipids in adipocytes that are further processed by the mitochondria during thermogenesis [19, 29]. Free fatty acid-driven oxidized electrons are carried through the mitochondrial ETC complexes and complemented by UCP1 to produce cellular ATP [29]. Active thermogenesis increases oxygen consumption and mitochondrial OXPHOS proteins [19]. In our study, *B. bifidum* DS0908 and *B. longum* DS0950 increased mitochondrial UCP1 and OXPHOS protein expressions. In addition, mitochondrial biogenesis increases mitochondrial content, which is known to contribute to thermogenesis. PGC1α reportedly improves mitochondrial biogenesis and oxidative phosphorylation, induced upon *B. bifidum* DS0908 and *B. longum* DS0950 supplementation [27, 28]. PRDM16 reportedly complements beige and brown adipocyte activity both in mice and humans [24]. The *Bifidobacterium* strains considered in the current study increased the PRDM16 level.

Mature white adipocytes are characterized by adipocyte-binding protein 4 (*aP2*), *Psat1*, *Resistin* and *Serpina3k*; beige and brown adipocytes, by *Cd137*, *Tbx1* and *Fgf21*, as well as *Cox2* and *P2rx5*, expressions, respectively [8]. Several studies described how an increase in beige and brown adipocyte marker expression reduces the levels of white adipocyte marker expression. We also observed the same expression patterns following *B. bifidum* DS0908 and *B. longum* DS0950 supplementation [8, 19]. The increased expression of beige and brown adipocyte markers implies thermogenic improvement which alleviates obesity. Several signaling pathways tightly regulate the induction of these beige and brown adipocyte signatures. During the browning process, therapeutic candidates promote signaling protein (*e.g.*, AMPK, PKA, p38 MAPK and CREB) phosphorylation, further inducing downstream thermogenic (common beige and brown) protein (*i.e.*, PPARγ, PRDM16, PGC1α and UCP1) expressions [8, 19, 21, 22]. Previous studies demonstrated that several probiotics, including *B. infantis*, can inhibit adiposity via AMPK and PKA/p38 MAPK signaling activation [7, 8, 44]. In addition, culture supernatants (postbiotics) produced by *B. longum* positively regulated thermogenesis via PKA signaling activation [8]. Isolated fatty acids (short-, medium- and long-chain fatty acids; postbiotics) from beneficial microbes (probiotics) positively affected thermogenic induction via GPR signaling induction. They reportedly alleviate obesity by inducing sustainable thermogenesis via GPR activation, such as GPR40, GPR43, GPR45 and GPR120 [10, 13, 47, 48]. Interestingly, fatty acids can penetrate the blood-brain barrier and modulate insulin and leptin receptor expression in the hypothalamus via GPR signaling, possibly improving sustainable energy metabolism [10, 13, 47, 48]. It is noteworthy that fatty acids activate cell surface GPR expression, further modulating the underlying signaling proteins (*e.g.*, PKA, P38 MAPK and AMPK) [49-51]. In our study, culture supernatants of *B. bifidum* DS0908 and *B. longum* DS0950 activated PKA/p38 MAPK signaling in C3H10T1/2 MSCs during the promotion of thermogenesis. It is worth mentioning that multiple studies have investigated crude culture supernatants of probiotics as viable components to understand their role in alleviating obesity [7, 8, 44]. Although our study provides evidence of the thermogenic effect of *B. bifidum* DS0908 and *B. longum* DS0950 pellets and culture supernatants both in vitro and in vivo, these findings could be firmly bolstered by identifying key metabolites and investigating their anti-obesity effects via browning activation.

In summary, our study provides in vitro and in vivo evidence that *B. bifidum* DS0908 and *B. longum* DS0950 pellets and culture supernatants ameliorate obesity by activating the thermogenic program. In vivo study revealed that they improved the plasma lipid profile, insulin sensitivity, and glucose metabolism without altering food intake, suggesting that *B. bifidum* DS0908 and *B. longum* DS0950 and their culture supernatants might mitigate the clinical onset of obesity.

## Supplemental Materials

Supplementary data for this paper are available on-line only at http://jmb.or.kr.

## Figures and Tables

**Table 1:** Primers used in this study.

Primer Name	Forward (5’-3’)	Reverse (5’-3’)
*mUcp1*	GGCATTCAGAGGCAAATCAGCT	CAATGAACACTGCCACACCTC
*mPrdm16*	CAGCACGGTGAAGCCATTC	GCGTGCATCCGCTTGTG
*mPgc1α*	ACAGCTTTCTGGGTGGATT	TGAGGACCGCTAGCAAGTTT
*mCd137*	CGTGCAGAACTCCTGTGATAAC	GTCCACCTATGCTGGAGAAGG
*mCox2*	GACTGGGCCATGGAGTGG	CACCTCTCCACCAATGACC
*mTbx1*	GGCAGGCAGACGAATGTTC	TTGTCATCTACGGGCACAAAG
*mFgf21*	AGATCAGGGAGGATGGAACA	TCAAAGTGAGGCGATCCATA
*mP2Rx5*	CTGCAGCTCACCATCCTGT	CACTCTGCAGGGAAGTGTCA
*maP2*	GTGATGCCTTTGTGGGAAACCTGGAAG	TCATAAACTCTTGTGGAAGTCACGCC
*mPparγ*	TTTGAAAGAAGCGGTGAACCAC	ACCATTGGGTCAGCTCTTGTG
*mPsat1*	TACCGCCTTGTCAAGAAACC	AGTGGAGCGCCAGAATAGAA
*mResistin*	TGCCAGTGTGCAAGGATAGACT	CGCTCACTTCCCCGACAT
*mSerpina3k*	GGCTGAAGGCAAAGTCAGTGT	TGGAATCTGTCCTGCTGTCCT
*mTbp*	GAAGCTGCGGTACAATTCCAG	CCCCTTGTACCCTTCACCAAT

## References

[1] Yoo JY, Kim SS (2016). Probiotics and prebiotics: Present status and future perspectives on metabolic disorders. Nutrients.

[2] George Kerry R, Patra JK, Gouda S, Park Y, Shin H-S, Das G (2018). Benefaction of probiotics for human health: a review. J. Food Drug Anal..

[3] Davani-Davari D, Negahdaripour M, Karimzadeh I, Seifan M, Mohkam M, Masoumi SJ (2019). Prebiotics: definition, types, sources, mechanisms, and clinical applications. Foods.

[4] Markowiak P, Śliżewska K (2017). Effects of probiotics, prebiotics, and synbiotics on human health. Nutrients.

[5] Sugahara H, Odamaki T, Fukuda S, Kato T, Xiao J-z, Abe F (2015). Probiotic *Bifidobacterium longum* alters gut luminal metabolism through modification of the gut microbial community. Sci. Rep..

[6] Reynés B, Palou M, Rodríguez AM, Palou A (2019). Regulation of adaptive thermogenesis and browning by prebiotics and postbiotics. Front. Physiol..

[7] Rahman MS, Kang I, Lee Y, Habib MA, Choi BJ, Kang JS (2021). *Bifidobacterium longum* subsp. *infantis* YB0411 inhibits adipogenesis in 3T3-L1 pre-adipocytes and reduces high-fat-diet-induced obesity in mice. J. Agric. Food Chem..

[8] Hossain M, Park DS, Rahman MS, Ki SJ, Lee YR, Imran KM (2020). *Bifidobacterium longum* DS0956 and *Lactobacillus rhamnosus* DS0508 culture-supernatant ameliorate obesity by inducing thermogenesis in obese-mice. Benef. Microbes..

[9] Hu J, Kyrou I, Tan BK, Dimitriadis GK, Ramanjaneya M, Tripathi G (2016). Short-chain fatty acid acetate stimulates adipogenesis and mitochondrial biogenesis via GPR43 in brown adipocytes. Endocrinology.

[10] Haynes VR, Michael NJ, van den Top M, Zhao FY, Brown RD, De Souza D (2020). A neural basis for octanoic acid regulation of energy balance. Mol. Metab..

[11] Cho Y, Shamim Rahman M, Kim Y-S (2019). Obesity regulation through gut microbiota modulation and adipose tissue browning. J. Life Sci..

[12] Chichlowski M, Shah N, Wampler JL, Wu SS, Vanderhoof JA (2020). *Bifidobacterium longum* subspecies infantis (*B. infantis*) in pediatric nutrition: current state of knowledge. Nutrients.

[13] Christian M (2020). Elucidation of the roles of brown and brite fat genes: GPR120 is a modulator of brown adipose tissue function. Exp. Physiol..

[14] Cypess AM, Kahn CR (2010). Brown fat as a therapy for obesity and diabetes. Curr. Opin. Endocrinol. Diabetes Obes..

[15] Zwick RK, Guerrero-Juarez CF, Horsley V, Plikus MV (2018). Anatomical, physiological, and functional diversity of adipose tissue. Cell Metab..

[16] Longo M, Zatterale F, Naderi J, Parrillo L, Formisano P, Raciti GA (2019). Adipose tissue dysfunction as determinant of obesityassociated metabolic complications. Int. J. Mol. Sci..

[17] Ouchi N, Parker JL, Lugus JJ, Walsh K (2011). Adipokines in inflammation and metabolic disease. Nat. Rev. Immunol..

[18] Rosenwald M, Wolfrum C (2014). The origin and definition of brite versus white and classical brown adipocytes. Adipocyte.

[19] Hossain M, Imran KM, Rahman MS, Yoon D, Marimuthu V, Kim YS (2020). Sinapic acid induces the expression of thermogenic signature genes and lipolysis through activation of PKA/CREB signaling in brown adipocytes. BMB Rep..

[20] Loft A, Forss I, Siersbæk MS, Schmidt SF, Larsen A-SB, Madsen JGS (2015). Browning of human adipocytes requires KLF11 and reprogramming of PPARγ superenhancers. Genes Dev..

[21] Rahman MS, Imran KM, Hossain M, Lee T-J, Kim Y-S (2021). Biochanin A induces a brown-fat phenotype via improvement of mitochondrial biogenesis and activation of AMPK signaling in murine C3H10T1/2 mesenchymal stem cells. Phytother. Res..

[22] Imran KM, Rahman N, Yoon D, Jeon M, Lee B-T, Kim Y-S (2017). Cryptotanshinone promotes commitment to the brown adipocyte lineage and mitochondrial biogenesis in C3H10T1/2 mesenchymal stem cells via AMPK and p38-MAPK signaling. Biochim. Biophys. Acta Mol. Cell Biol. Lipids.

[23] Tzameli I, Fang H, Ollero M, Shi H, Hamm JK, Kievit P (2004). Regulated production of a peroxisome proliferator-activated receptor-γ ligand during an early phase of adipocyte differentiation in 3T3-L1 adipocytes. J. Biol. Chem..

[24] Becerril S, Gómez-Ambrosi J, Martín M, Moncada R, Sesma P, Burrell MA (2013). Role of PRDM16 in the activation of brown fat programming. Relevance to the development of obesity. Histol. Histopathol..

[25] Wang W, Ishibashi J, Trefely S, Shao M, Cowan AJ, Sakers A (2019). A PRDM16-driven metabolic signal from adipocytes regulates precursor cell fate. Cell Metab..

[26] Ishibashi J, Seale P (2015). Functions of *Prdm16* in thermogenic fat cells. Temperature (Austin).

[27] Ventura-Clapier R, Garnier A, Veksler V (2008). Transcriptional control of mitochondrial biogenesis: the central role of PGC-1α. Cardiovasc. Res..

[28] Cheng C-F, Ku H-C, Lin H (2018). PGC-1α as a pivotal factor in lipid and metabolic regulation. Int. J. Mol. Sci..

[29] Smith RAJ, Hartley RC, Cochemé HM, Murphy MP (2012). Mitochondrial pharmacology. Trends Pharmacol. Sci..

[30] Coleman OI, Haller D (2017). Bacterial signaling at the intestinal epithelial interface in inflammation and cancer. Front. Immunol..

[31] Mishra A, Lai GC, Yao LJ, Aung TT, Shental N, Rotter-Maskowitz A (2021). Microbial exposure during early human development primes fetal immune cells. Cell.

[32] Kim JS, Choe H, Kim KM, Lee YR, Rhee MS, Park DS (2018). *Lactobacillus porci* sp. nov., isolated from small intestine of a swine. Int. J. Syst. Evol. Microbiol..

[33] Prasad J, Gill H, Smart J, Gopal PK (1998). Selection and characterisation of *Lactobacillus* and *Bifidobacterium* strains for use as probiotics. Int. Dairy J..

[34] Heymsfield SB, Wadden TA (2017). Mechanisms, pathophysiology, and management of obesity. New Eng. J. Med..

[35] Petrakis D, Margină D, Tsarouhas K, Tekos F, Stan M, Nikitovic D (2020). Obesity - a risk factor for increased COVID-19 prevalence, severity and lethality (Review). Mol. Med. Rep..

[36] Vallianou N, Stratigou T, Christodoulatos GS, Tsigalou C, Dalamaga M (2020). Probiotics, prebiotics, synbiotics, postbiotics, and obesity: current evidence, controversies, and perspectives. Curr. Obes. Rep..

[37] Rosen ED, Walkey CJ, Puigserver P, Spiegelman BM (2000). Transcriptional regulation of adipogenesis. Genes Dev..

[38] Mota de Sá P, Richard AJ, Hang H, Stephens JM (2017). Transcriptional regulation of adipogenesis. Comp. Physiol..

[39] Rajesh Y, Sarkar D (2021). Association of adipose tissue and adipokines with development of obesity-induced liver cancer. Int. J. Mol. Sci..

[40] Deng Y, Scherer PE (2010). Adipokines as novel biomarkers and regulators of the metabolic syndrome. Ann. N Y Acad. Sci..

[41] An HM, Park SY, Lee DK, Kim JR, Cha MK, Lee SW (2011). Antiobesity and lipid-lowering effects of *Bifidobacterium* spp. in high fat diet-induced obese rats. Lipids Health Dis..

[42] Arias-Mutis OJ, Marrachelli VG, Ruiz-Sauri A, Alberola A, Morales JM, Such-Miquel L (2017). Development and characterization of an experimental model of diet-induced metabolic syndrome in rabbit. PLoS One.

[43] Liu Y, Gao Y, Ma F, Sun M, Mu G, Tuo Y (2020). The ameliorative effect of *Lactobacillus plantarum* Y44 oral administration on inflammation and lipid metabolism in obese mice fed with a high fat diet. Food Funct..

[44] Soundharrajan I, Kuppusamy P, Srisesharam S, Lee JC, Sivanesan R, Kim D (2020). Positive metabolic effects of selected probiotic bacteria on diet-induced obesity in mice are associated with improvement of dysbiotic gut microbiota. FASEB J..

[45] Stine RR, Shapira SN, Lim HW, Ishibashi J, Harms M, Won KJ (2016). EBF2 promotes the recruitment of beige adipocytes in white adipose tissue. Mol. Metab..

[46] Lee P, Werner CD, Kebebew E, Celi FS (2014). Functional thermogenic beige adipogenesis is inducible in human neck fat. Int. J. Obes..

[47] Miyamoto J, Hasegawa S, Kasubuchi M, Ichimura A, Nakajima A, Kimura I (2016). Nutritional signaling via free fatty acid receptors. Int. J. Mol. Sci..

[48] Ichimura A, Hasegawa S, Kasubuchi M, Kimura I (2014). Free fatty acid receptors as therapeutic targets for the treatment of diabetes. Front. Pharmacol..

[49] Wang A, Si H, Liu D, Jiang H (2011). Butyrate activates the cAMP-protein kinase A-cAMP response element-binding protein signaling pathway in Caco-2 cells. J. Nutr..

[50] Wauson EM, Dbouk HA, Ghosh AB, Cobb MH (2014). G protein-coupled receptors and the regulation of autophagy. Trends Endocrinol. Metab..

[51] Barella LF, Jain S, Kimura T, Pydi SP (2021). Metabolic roles of G protein-coupled receptor signaling in obesity and type 2 diabetes. FEBS J..

