# Supplementary material for: Bifidobacterium bifidum DS0908 and Bifidobacterium longum DS0950 Culture-Supernatants Ameliorate Obesity-Related Characteristics in Mice with High-Fat Diet-Induced Obesity
Source: J Microbiol Biotechnol. 2022 Nov 22;33(1):96–105. doi: 10.4014/jmb.2210.10046 (PMC9899789; doi:10.4014/jmb.2210.10046)
Supplement: Supplementary file 1 [file jmb-33-1-96-supple.pdf]

J. Microbiol. Biotechnol.
https://doi.org/10.4014/jmb.2210.10046

Supplementary Materials

Bifidobacterium bifidum DS0908 and *Bifidobacterium longum* DS0950 culture-supernatants ameliorate obesity-related characteristics in mesenchymal stem cells and mice with high-fat diet-induced obesity

MS Rahman[1, 2, #], Y Lee[1, 2, #], DS Park[3], and YS Kim [1, 2, *]

[1] Institute of Tissue Regeneration, College of Medicine, Soonchunhyang University, Cheonan, Chung-nam 31151, Republic of Korea

[2] Department of Microbiology, College of Medicine, Soonchunhyang University, Cheonan, Chung-nam 31151, Republic of Korea

[3] Biological Resource Center, Korea Research Institute of Bioscience and Biotechnology, Jeongeup, Republic of Korea

[#] These authors contributed equally

* Corresponding author:

Yong-Sik Kim, Ph.D.

Email: yongsikkim@sch.ac.kr

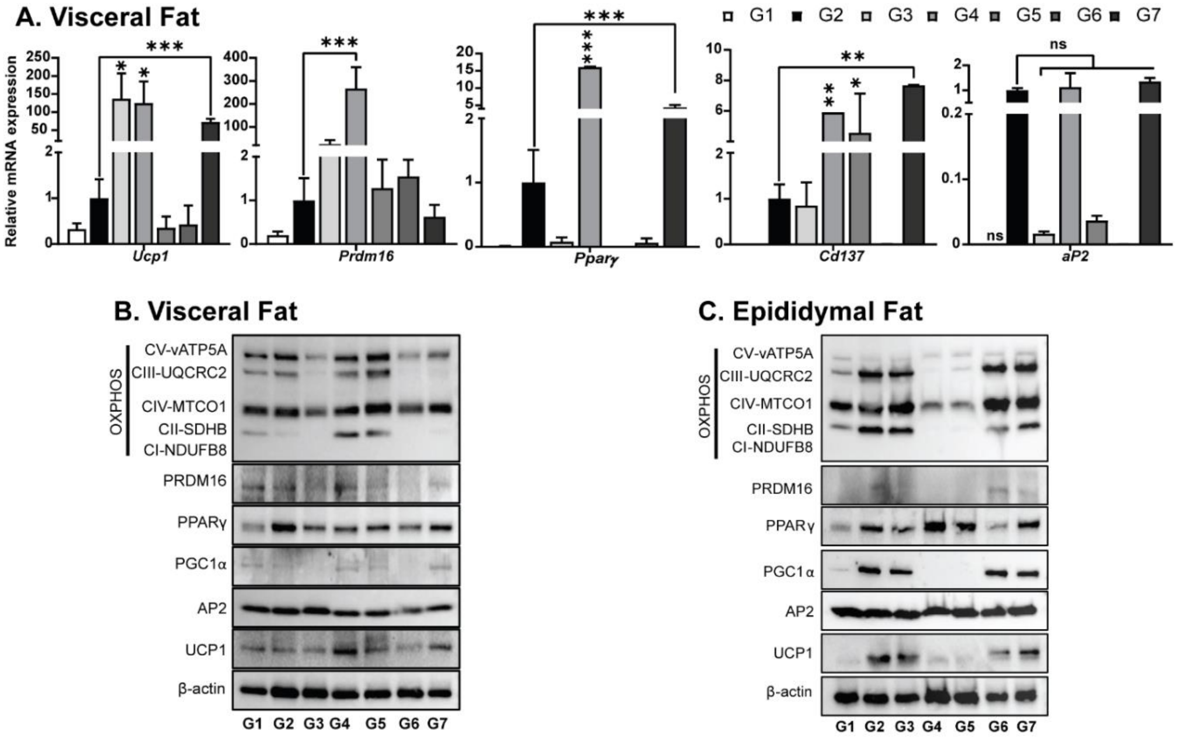

Figure S1. Effect of DS0908 and DS0950 administration on the expression of brown adipocyte-specific markers in mice with HFD-induced obesity. (A) *Ucp1, Prdm16, Pparγ, Cd137* and *aP2* mRNA expression levels. (B and C) UCP1, AP2, PGC-1α, PPARγ, PRDM16 and mitochondrial oxidative phosphorylation protein expression levels in the visceral and epididymal fat of DS0908 and DS0950-administered mice with HFD-induced obesity. *Tbp* was used as an internal control gene and β- actin as a protein loading control. The data from three individual experiments are expressed as the average ± standard error mean (SEM). *, **, *** and ns indicate $p < 0.05$, < 0.01, < 0.001 and non-significant, respectively, to express the statistically significant differences between the control (G2: HFD) and treatment groups. G1: Normal-fat diet (NFD); G2: High-fat diet (HFD); G3: BS DS0908 (DS0908); G4: BS DS0950

(DS0950); G5: BP DS0908 (DS0908; 10^9 cells/kg); G6: BP DS0950 (DS0950; 10^9 cells/kg); G7: Rosiglitazone (Rosi; 10 mg/kg); BS = bacterial supernatant; BP = bacterial pellets.

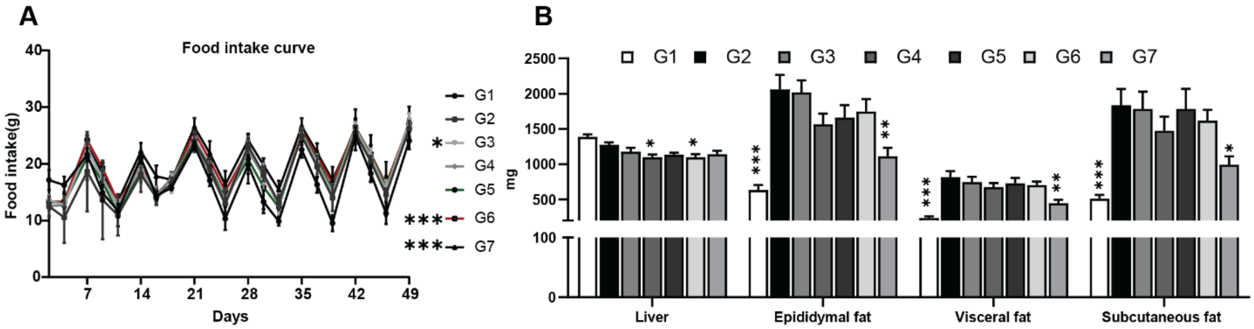

Figure S2. DS0908 and DS0950 supplementation reduces organ weight in mice with HFD-induced obesity. (A) Food intake changes in DS0908- and DS0950-administered mice with HFD-induced obesity. (B) Organ weight measurements (epididymal, visceral and subcutaneous fat, as well as liver). The data are expressed as the average ± standard error mean (SEM). *, **, *** and ns indicate $p < 0.05$, < 0.01, < 0.001 and non-significant, respectively, to express the statistically significant differences between the control (G2: HFD) and treatment groups. G1: Normal-fat diet (NFD); G2: High-fat diet (HFD); G3: BS DS0908 (DS0908); G4: BS DS0950 (DS0950); G5: BP DS0908 (DS0908; 10^9 cells/kg); G6: BP DS0950 (DS0950; 10^9 cells/kg); G7: Rosiglitazone (Rosi; 10 mg/kg); BS = bacterial supernatant; BP = bacterial pellets.

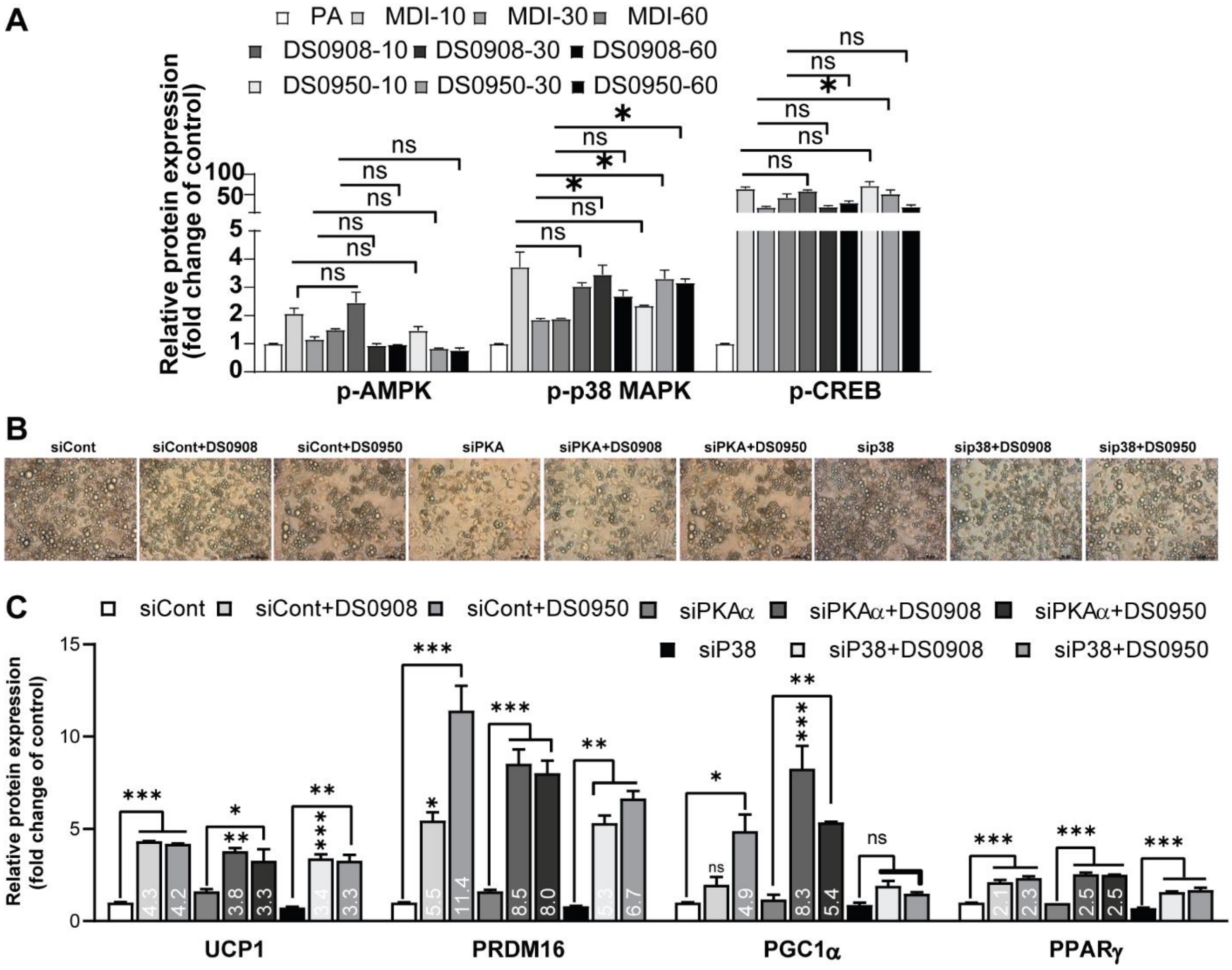

Figure S3. DS0908 and DS0950 culture-supernatants activate thermogenesis via PKA-p38 MAPK signaling in C3H10T1/2 MSCs. (A) Phosphorylated protein expression levels of p-p38 MAPK, p-CREB and p-AMPK after incubation with DS0908 and DS0950 for 10, 30 and 60 min. (B) Microscopic images of lipid droplet in *Pkaα* and *p38 MAPKα*-knockdown cells, and treatment with DS0908 and DS0950 (siPkaα/sip38 MAPKα + DS0908 or DS0950 group) and control siRNA and treatment with DS0908 or DS0950 (siCont + DS0908 or DS0950 group). (C) Protein expression levels (UCP1, PGC1α, PRDM16 and PPARγ) were measured after silencing of *Pkaα* and *p38 MAPKα* and treatment with DS0908 or DS0950 (siPkaα/sip38 MAPKα +

DS0908 or DS0950 group) and control siRNA and treatment with DS0950 or DS0908 (siCont + DS0908 or DS0950 group). The gene knockdown experiments were designed as siCont vs. siCont + DS0908 or DS0950, siPkaα vs. siPkaα + DS0908 or DS0950 and sip38 MAPKα vs. sip38 MAPKα + DS0908 or DS0950. The protein expression band intensities were measured by with ImageJ. The data from three individual experiments are expressed as the average ± standard error mean (SEM). *, **, *** and ns indicate $p < 0.05$, < 0.01, < 0.001 and non-significant, respectively, to express the statistically significant differences between the control (MDI) and the treatment groups in the figures. Adipogenic differentiation medium, MDI: 0.5 mM IBMX, 1 μM dexamethasone and 10 μg/mL insulin; 1 μM Rosiglitazone (Rosi); DS0908 = *B. bifidum* DS0908; DS0950 = *B. longum* DS0950.

Supplementary Table 1. List of probiotic bacterial strains screened for anti-obesity effect.

Serial No.	Strains	Isolation No.	Strain No.	Origin	Source
1	*B. bifidum*	01S5		Infant	faeces
2	*B. breve*	01B6		Infant	faeces
3	*B. breve*	01B8		Infant	faeces
4	*B. bifidum*	02S3		Infant	faeces
5	*B. bifidum*	02S5		Infant	faeces
6	*B. bifidum*	02S8		Infant	faeces
7	*B. bifidum*	02S18		Infant	faeces
8	*B. longum*	05S61		Infant	faeces
9	*B. breve*	05S71		Infant	faeces
10	*B. breve*	05S76		Infant	faeces
11	*B. longum*	05B23		Infant	faeces
12	*B. longum*	06S63		Infant	faeces
**13**	***B. bifidum***	**07S4**	**DS0908 (HN002)**	**Infant**	**faeces**
14	*B. longum*	07S5		Infant	faeces
15	*B. bifidum*	07S8		Infant	faeces
16	*B. bifidum*	07S13		Infant	faeces
17	*B. longum*	07S14		Infant	faeces
18	*B. longum*	07S15		Infant	faeces
19	*B. bifidum*	07S25		Infant	faeces
20	*B. bifidum*	07S26		Infant	faeces
21	*B. bifidum*	07S29		Infant	faeces
22	*B. longum*	07S35		Infant	faeces
23	*B. longum*	07S37		Infant	faeces
24	*B. longum*	07S38		Infant	faeces
25	*B. longum*	07S41		Infant	faeces
26	*B. longum*	08S6		Infant	faeces
27	*B. longum*	08S18		Infant	faeces
**28**	***B. longum***	**08S22**	**DS0950 (HN001)**	**Infant**	**faeces**
29	*B. longum*	08S40		Infant	faeces
30	*B. longum*	08S41	DS0956	Infant	faeces
31	*B. longum*	08S45		Infant	faeces
32	*B. longum*	08S46		Infant	faeces
33	*B. longum*	08S62		Infant	faeces
34	*B. longum*	08S643		Infant	faeces
35	*B. animalis*	08S70		Infant	faeces
36	*L. reuteri*	MBF4116		Infant	faeces
37	*L. reuteri*	AN417		Infant	faeces
38	*L. rhamnosus*	B1		Infant	faeces
39	*L. rhamnosus*	OMM118		Infant	faeces

40	*L. rhamnosus*	OMM228		Infant	faeces
41	*L. rhamnosus*	B1S1		Infant	faeces
42	*L. rhamnosus*	B2S1		Infant	faeces
43	*L. gasseri*	MBF402		Infant	faeces
44	*L. gasseri*	MF203		Infant	faeces
45	*L. gasseri*	NM518		Infant	faeces
46	*L. gasseri*	MM332		Infant	faeces
47	*L. gasseri*	SM353		Infant	faeces
48	*L. acidophilus*	C48		Infant	faeces
49	*L. acidophilus*	S42		Infant	faeces
50	*L. rhamnosus*	B15		Infant	faeces
51	*L. rhamnosus*	OMM135	DS0508	Infant	faeces
52	*L. delbrueckii*	AN315		Infant	faeces
53	*L. delbrueckii*	AN617		Infant	faeces
54	*L. fermentum*	C62		Infant	faeces
55	*L. fermentum*	SBF308		Infant	faeces
